# Hidden by bias: how standard psychophysical procedures conceal crucial aspects of peripheral visual appearance

**DOI:** 10.1038/s41598-021-83325-7

**Published:** 2021-02-18

**Authors:** Fazilet Zeynep Yildirim, Daniel R. Coates, Bilge Sayim

**Affiliations:** 1 Institute of Psychology, University of Bern, Fabrikstrasse 8, 3012 Bern, Switzerland; 2 College of Optometry, University of Houston, Houston, TX 77204 USA; 3 SCALab-Sciences Cognitives et Sciences Affectives, CNRS, UMR 9193, University of Lille, 59000 Lille, France

**Keywords:** Human behaviour, Perception

## Abstract

The perception of a target depends on other stimuli surrounding it in time and space. This contextual modulation is ubiquitous in visual perception, and is usually quantified by measuring performance on sets of highly similar stimuli. Implicit or explicit comparisons among the stimuli may, however, inadvertently bias responses and conceal strong variability of target appearance. Here, we investigated the influence of contextual stimuli on the perception of a repeating pattern (a line triplet), presented in the visual periphery. In the *neutral* condition, the triplet was presented a single time to capture its minimally biased perception. In the *similar* and *dissimilar* conditions, it was presented within stimulus sets composed of lines similar to the triplet, and distinct shapes, respectively. The majority of observers reported perceiving a line pair in the *neutral* and *dissimilar* conditions, revealing ‘redundancy masking’, the reduction of the perceived number of repeating items. In the *similar* condition, by contrast, the number of lines was overestimated. Our results show that the *similar* context did not reveal redundancy masking which was only observed in the *neutral* and *dissimilar* context. We suggest that the influence of contextual stimuli has inadvertently concealed this crucial aspect of peripheral appearance.

## Introduction

Visual targets are rarely seen in isolation, as they are usually surrounded by contextual stimuli in space and time. The spatial and temporal contexts often strongly influence how a target is perceived^1–9^. For example, in crowding, flanking items deteriorate performance on a target that is easily identified in isolation^10–19^. In the temporal domain, contextual influences such as set size^2,3^ and stimulus range^2–4,20,21^ have been shown to modulate target processing, with processing speed of targets depending on the size and the familiarity of the stimulus set in which the targets were embedded^22,23^. For example, reaction times for indicating target presence/absence were faster when the stimulus set contained only two compared to eight different items^22^. Similarly, the distribution and range of contextual stimuli influence perceptual judgments^2–4,20,21^. For example, it was shown that stimulus distributions systematically biased estimates of the length of time intervals toward the mean of the underlying distribution^21^. As stimuli in standard psychophysical experiments are usually presented in blocks with other stimuli, different stimulus sets may thus inadvertently influence target perception.

Contextual stimuli in the temporal domain may exert their influence on target perception over different periods of time, with effects lasting from only up to a few trials^24–27^ to longer periods of several minutes (e.g.^28^). These influences manifest as systematic biases. In particular, judgments of the current stimulus were shown to be attracted by stimuli in the immediately preceding trial, and repelled by stimuli in the two to five trials before (e.g.^2^). While such contextual influences have beneficial effects, for example, by stabilizing perception, the introduced biases may conceal important aspects of target appearance. Here, we show how contextual influences affect the perception in stimuli susceptible to ‘redundancy masking’, the recently discovered phenomenon that entire items within repeating patterns are not reported^29,30^.

**Figure 1:**
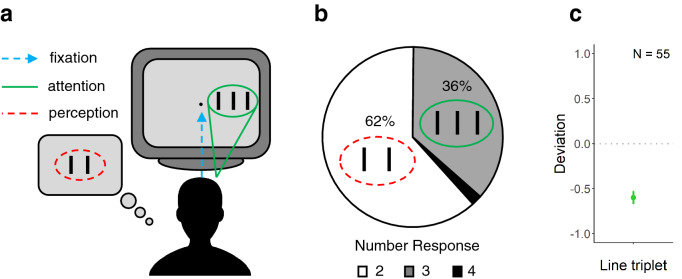
Illustration of redundancy masking (**a**), and results of Experiment 1 (**b**,**c**). (**a**) While fixating on the center (blue dashed arrow), observers attended to the stimulus in the periphery. When presented with three lines, most observers reported two lines. (**b**) Percentages of perceived numbers of lines. 61.8% of the observers reported perceiving a line pair, 36.4% reported a line triplet, and 1.8% reported a line quadruplet. (**c**) The average deviation score for the line triplet. Deviation scores were calculated by subtraction of the number of lines presented from the number of lines reported. Error bar shows ± *SEM*.

In redundancy masking, redundant elements in displays containing as few as three repeating elements are unavailable for conscious report (Fig. 1a^29,30^). For example, when asked to indicate the number of three closely-spaced identical elements presented in the visual periphery, the majority of observers reported only two elements (Fig. 1a^29,30^). Redundancy masking has been shown for simple stimuli such as lines^30^ as well as more complex stimuli (letters^29^). It resembles visual crowding to some extent but differs in regard to a number of key characteristics^30,31^. Both redundancy masking and crowding are stronger when the items are near each other^10^, radially arranged^11^, grouped together^15–17^, and similar^32,33^. Although crowding is usually stronger when a target is flanked by similar items^32,33^, the strength of target disruption in conditions where the flankers are the same as the target is poorly understood. On the one hand, it was suggested that identification performance for the central target is better when all items are the same^34,35^. On the other hand, it was argued that a bias to respond with the identity of the flankers could explain seemingly superior performance with identical items in typical identification experiments^29,36,37^. A typical letter identification paradigm revealed very good performance when all letters of a trigram were identical, whereas asking observers to freely report and draw what they saw revealed strong redundancy masking: most observers reported only two letters^29^. These findings may partly explain that despite a plethora of crowding experiments in which small numbers of often highly similar items (e.g., letter trigrams) were presented, the effect of redundancy masking has not been reported until recently. In typical crowding tasks, observers are usually not asked to report the number of items because failures to detect the target is not expected as crowding is assumed to spare detection^12,14^. Hence, it remained unclear how many items participants actually perceived in these tasks. Here, we investigated to what extent the types of contextual stimuli used in psychophysical experiments have contributed to the elusiveness of redundancy masking, and show how different sets of contextual stimuli determine the presence or absence of a strong effect that, we propose, is a fundamental characteristic of peripheral vision.

In all experiments, the main target consisted of three peripherally presented, closely spaced lines (line triplet). The target was presented in blocks containing different contextual stimuli. There were three conditions: neutral, similar, and dissimilar context. In the *neutral* condition, the aim was to minimize systematic effects of contextual stimuli on target perception by preventing comparisons with any other stimuli: In a single trial paradigm, a large number of observers were presented with the line triplet once, and asked to freely report what they perceived. The majority of observers (62%) reported perceiving two lines (average number of lines perceived = 2.40), showing strong redundancy masking. In the *similar* condition, the line triplet was presented among similar contextual stimuli (like those often used in psychophysical tasks), consisting of 1 to 5 lines. Observers were asked to report the number of lines. In contrast to the *neutral* condition, observers mostly reported three or more lines (M = 3.37) when presented with the line triplet. In the *dissimilar* condition, the line triplet was presented among dissimilar contextual stimuli consisting again of 1 to 5 items. The items were different in shape and spatial arrangement from the line triplet. Observers indicated the number of items. As in the *neutral* condition, we found strong redundancy masking for the line triplet (M = 2.31). These results suggest that the appearance in the *similar* context strongly deviated from the appearance in the *neutral* and *dissimilar* context conditions. We suggest that the characteristics of contextual stimuli can inadvertently affect perception in standard psychophysical paradigms and conceal crucial aspects of appearance.

## Results

### Experiment 1: neutral context

The aim of the first experiment was to capture how the line triplet was perceived in the absence of a contextual stimulus set. For this aim, in a single trial paradigm, observers were presented with the line triplet centered at 10 degrees in the periphery a single time.

61.8% of the observers reported perceiving a line pair, 36.4% a line triplet, and 1.8% a line quadruplet (Fig. 1b). The sign test showed that the number of lines reported was lower than the number of lines presented (Fig. 1c: average deviation score = − 0.60 ± *SD* 0.53, *Z* = − 5.41, *p* < 0.00001), showing strong redundancy masking. This result shows how the line triplet was perceived when not influenced by contextual stimuli, revealing minimally biased (or unbiased) perception.

### Experiment 2: similar context

**Figure 2:**
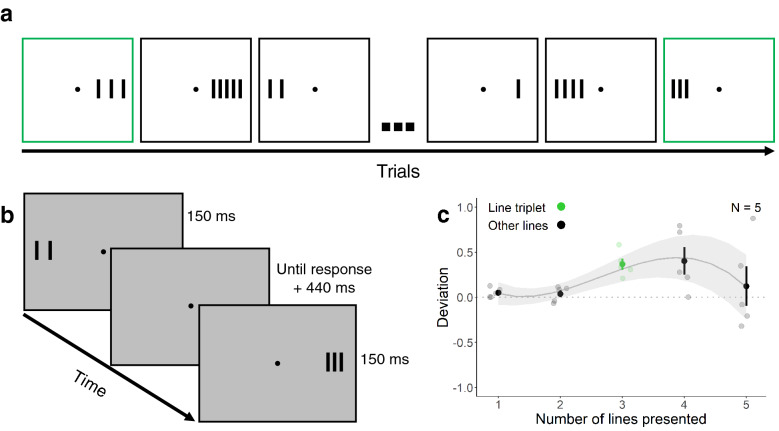
Illustration of a sequence of trials (**a**), schematic depiction of the experimental paradigm (**b**), and results of Experiment 2 (**c**). (**a**) The line triplet is shown with green frames (for illustration). (**b**) Observers were randomly presented with 1 to 5 lines with different spacings to the left or right side of fixation. (**c**) Deviation scores are plotted as a function of the number of lines presented. Black and green data points show mean deviation scores (± *SEM*). Grey and light green data points show mean deviation scores for each individual observer. The grey line and shaded regions show the model fit and confidence intervals (± 1.96 * *SEM*).

In the *similar* context experiment, we presented the line triplet in blocks with similar line stimuli of different numbers (1 to 5 lines; Fig. 2a,b). We expected that because of implicit or explicit comparisons between similar stimuli, including stimuli that were less ambiguous than the line triplet (single lines and pairs of lines), no redundancy masking would occur.

Figure 2c shows the deviation scores for Experiment 2 as a function of the number of lines presented. The number of lines reported was slightly greater than the number of lines presented (average deviation scores: single line, 0.05 ± *SD* 0.05; line pair, 0.04 ± *SD* 0.08; line triplet, 0.37 ± *SD* 0.14; line quadruplet, 0.40 ± *SD* 0.34; line quintuplet, 0.12 ± *SD* 0.49). There was higher overestimation of the number of lines with the smallest spacing (0.39°) for 4 and 5 lines (2 and 3 lines were not significantly affected by the spacing although there was a similar trend for 3 lines); however, none of the spacings yielded notable underestimation (for the spacings of 0.39°, 058°, and 0.78°, respectively: 2 lines: 0.05 ± *SD* 0.08, − 0.05 ± *SD* 0.26, 0.11 ± *SD* 0.09; 3 lines 0.57 ± *SD* 0.18, 0.36 ± *SD* 0.19, 0.18 ± *SD* 0.17; 4 lines: 0.71 ± *SD* 0.67, 0.28 ± *SD* 0.23, 0.22 ± *SD* 0.22; 5 lines: 0.45 ± *SD* 0.95, 0.06 ± *SD* 0.38, − 0.14 ± *SD* 0.20). A one-sample t-test to assess if responses to the line triplet deviated from presented number of lines showed that the number of lines reported was greater than the number of lines presented (*t*(4) = 5.87, *p* = 0.004, *d* = 2.62).

As opposed to the neutral condition where 62% of the subjects perceived a line pair (average deviation score: − 0.60 ± *SD* 0.53), the line triplet was perceived as a line pair only in 6% of the trials (average deviation score: 0.37 ± *SD* 0.14; see above). We suggest that because of implicit or explicit comparisons with other numbers of lines, in particular, the relatively more distinct single lines and line pairs, observers did not report a line pair when presented with the line triplet. These results show that presenting a stimulus in a context consisting of similar stimuli can strongly influence perception: Instead of redundancy masking as observed in Experiment 1 (neutral context), Experiment 2 showed an overestimation of the number of lines perceived when observers were presented with the line triplet.

In addition to the overall comparison between the different numbers of lines, we also examined if there was an effect of preceding trials on subsequent trials. We found an effect of stimuli presented one trial before the current trial (*X*^2^ (2) = 30.51, *p* = 2.376e−07; Supplementary Fig. S1). The number of lines reported was attracted towards the number of lines presented in one trial before. Importantly, regardless of the number of lines in the preceding trial, the average deviation score for the line triplet was always positive.

### Experiment 3: dissimilar context

**Figure 3:**
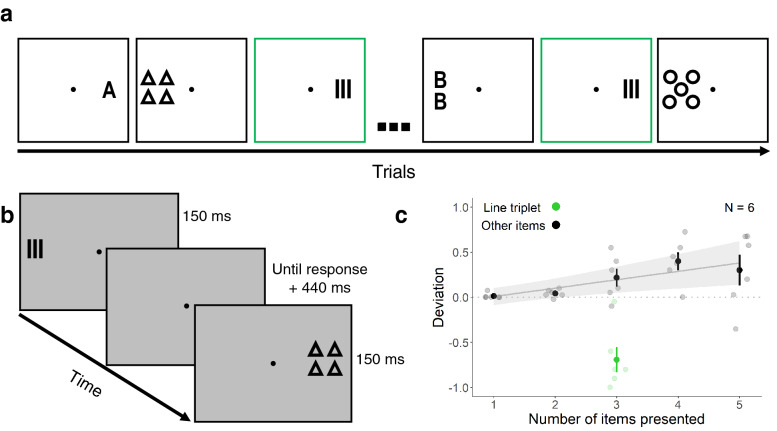
Illustration of a sequence of trials (**a**), schematic depiction of the experimental paradigm (**b**), and results of Experiment 3 (**c**). (**a**) The line triplet is shown with green frames (for illustration). (**b**) Observers were randomly presented with 1 to 5 items with different shapes to the left or right side of fixation. (**c**) Deviation scores are plotted as a function of the number of items presented. Black and green data points show mean deviation scores (± *SEM*). Grey and light green data points show mean deviation scores for each individual observer. The grey line and shaded regions show the model fit and confidence intervals (± 1.96 * *SEM*).

Experiment 2 showed that the similar stimulus context completely abolished the effect of redundancy masking: instead, the line triplet was perceived to contain more than three lines (overestimation). We suggest that this finding is likely a result of implicit or explicit comparisons between stimuli with similar characteristics. For example, comparing the line triplet with a perceptually relatively clearer pair of lines could help alleviate redundancy masking. If so, preventing such comparisons should result in a less biased or unbiased perception of the triplet (as in Experiment 1). To test this, the line triplet was presented in blocks consisting of a stimulus set with different shapes, hindering useful comparisons of the line triplet (Fig. 3a,b). The numbers of presented items were the same as in Experiment 2, varying between 1 and 5. We expected that impeding the possibility of direct comparisons with similar stimuli would yield redundancy masking for the line triplet.

Results for Experiment 3 are shown in Fig. 3c. The number of items reported was slightly greater than the number of items presented (average deviation scores: one item, 0.01 ± *SD* 0.03; two items, 0.04 ± *SD* 0.04; three items, 0.22 ± *SD* 0.24; four items, 0.40 ± *SD* 0.25; five items, 0.30 ± *SD* 0.42). The deviation score for the line triplet was − 0.69 ± *SD* 0.34, showing clear redundancy masking. A one-sample t-test for the line triplet showed that the number of lines reported was lower than the number of lines presented (*t*(5) = − 4.97, *p* = 0.004, *d* = − 2.03).

In contrast to the *similar* context condition, the line triplet presented in a set of dissimilar stimuli was predominantly perceived as a line pair. This finding indicates that as in the *neutral* context condition, the perception of the line triplet was minimally biased when it was presented in blocks with dissimilar contextual stimuli. This result strengthens the suggestion that comparisons between similar stimuli prevented redundancy masking.

As in Experiment 2, there was a positive effect of previous trials (i.e., one trial back) on subsequent trials, however, it only occurred for the line triplet (the line triplet: *X*^2^ (1) = 7.00, *p* = 0.0081; other items: *X*^2^ (1) = 1.63, *p* = 0.20; Supplementary Fig. S2). In contrast to the positive deviations scores in Experiment 2, the average deviation scores for the line triplet were negative for all numbers of lines in preceding trials.

## Discussion

Psychophysical experiments commonly measure performance by varying a single stimulus attribute on a given dimension. Here, we showed how the type of contextual stimuli that were presented in temporal proximity to a target stimulus either revealed or concealed the redundancy masking effect, the loss of stimulus elements in repeating patterns^29,30^. The target stimulus (the line triplet) was presented within a *neutral*, *dissimilar*, and *similar* stimulus context. In the *neutral* condition, the line triplet was presented in a single trial paradigm, and observers were asked to verbally report what they perceived. The majority of observers reported a line pair, revealing the recently discovered phenomenon of redundancy masking. When the line triplet was presented in a *similar* context where all contextual stimuli consisted of line arrays resembling the line triplet, no redundancy masking occurred. The *dissimilar* context condition was the same as the *similar* context condition except for the type of contextual stimuli which consisted of clusters of different shapes matching the numbers of presented lines in the *similar* context condition. Similar to the *neutral* condition, we found strong redundancy masking in the *dissimilar* context condition.

Our results show how the standard psychophysical procedure of presenting a range of highly similar stimuli completely concealed redundancy masking. Unlike previous studies showing effects of contextual stimuli^2,4,20,21^, we show that *similar* contextual stimuli resulted in a qualitatively different effect than the *neutral* and *dissimilar* context conditions. Hence, if the results of the *similar* condition were taken as an accurate approximation of how the line triplet was perceived, any conclusions would markedly differ from those drawn from the results of the *neutral* and *dissimilar* conditions. In particular, as the results of the *similar* condition did not reveal any redundancy masking but an overestimation of the number of lines, assumptions about characteristics of the peripheral visual system would need to incorporate a tendency for overestimation of small numbers of identical items. By contrast, the experiments with *neutral* and *dissimilar* contexts showed that the line triplet was mostly perceived as a line pair, showing the opposite effect—underestimation instead of overestimation. The conclusion that such target ‘diminishment’ is a characteristics of peripheral vision is in line with previous studies that investigated crowding^38–42^ (for differences between crowding and redundancy masking, see^29–31^).

We observed strong redundancy masking with the line triplet. Reporting only two items when three items were presented is uncommon and in contrast to the usual finding that the enumeration of small numbers of items is highly accurate. A key difference between larger (> 3) and smaller (≤ 3) numbers of items is that the number of the former needs to be estimated while the latter is usually ‘subitized’ (or ‘seen’) without error-prone estimation^43,44^. The difference between subitizing and estimation is usually also reflected in the variability of responses (smaller in subitizing and larger in estimation) which also seems to be the case for our findings (see Figs. 2c and 3c). This variability was revealed as overestimation in larger numbers in both experiments. More specifically, In Experiment 2, the number of lines reported varied with the number of lines presented in a non-monotonic manner, overall showing overestimation of the number. An initial negative slope of the deviation scores was followed by a positive slope, indicating increasing overestimation at mid-range (3 and 4), and then again by a negative slope, approaching accurate estimations at the largest number (5). In Experiment 3, the relationship between the deviation scores and the number of items presented (excluding the line triplet) was adequately captured by a linear model: overestimation increased with the number of items presented. The observed overestimation (for 3, 4, and 5 lines) cannot be explained by using the horizontal extent of the line array as a cue for the enumeration of the number of lines. For example, the 4-lines with the spacings 0.39° and 0.78° have the same extent as the 3-lines with spacing 0.58° and the 5-lines with spacing 0.58°, respectively. If responses were based on the extent of the arrays, observers should have overestimated larger spacings (0.78°) more than smaller spacings (0.39°). However, the results showed the opposite: observers overestimated the number of lines for small spacings and approached the correct estimation with larger spacings. Thus, we can exclude that the observed overestimation was due to observers’ using the overall extent of the line array as a cue.

We suggest that the overestimation in the *similar* condition is due to comparisons between similar stimuli, yielding the opposite of redundancy masked percept observed in the *neutral* and *dissimilar* conditions. An efficient way to overcome such contextual effects is to use a single trial paradigm^45–47^ (see also^48^). Although single trials cannot exclude all contextual factors that might influence observers’ judgments, they often provide accurate estimates of the probed feature, and may yield less biased responses than typical psychophysical paradigms that present several exemplars of similar stimuli^45,46^ (see also^9^, for a detailed discussion on single trials). Here, we used a single trial paradigm as one way to assess minimally biased appearance of the line triplet, and found strong redundancy masking. Attentional demands in the single trial paradigm could have played a role in the observed underestimation as participants did not know the exact location of the upcoming stimulus. However, we can exclude that insufficient attention explains the observed underestimation of the number of lines in the line triplet because the *dissimilar* context condition yielded the very same underestimation despite attentional demands resembling those in the *similar* condition. Moreover, strong redundancy masking was previously shown when viewing time was unconstrained and, hence, attentional constraints regarding the stimulus location did not play any role^29^.

The similarity between the target and contextual stimuli has been shown to be a decisive factor to invoke comparative judgments^3,49–55^. For example, objects that belong to the same category (e.g., two circles) are more readily compared than objects that belong to dissimilar categories (e.g., circles and squares^51,52^). In Experiment 2, the target line triplet and the contextual stimuli (1–5 lines) shared the same category (lines). No redundancy masking, but an overestimation of the number of lines in the line triplet was observed. We suggest that this result was due to implicit or explicit comparisons between the different numbers of lines in the stimulus set. In particular, the perceptually clearer pairs of lines (and single lines) may have served as ‘anchors’^5,56–58^ that biased observers and made them rarely report two lines (6% of the trials) when the line triplet was presented, but rather report three (58%) or four (30%) lines. That the absence of redundancy masking is due to the presentation of single lines and line pairs—and not lines per se—is supported by our previous findings of strong redundancy masking when observers were presented with 3–7 lines^30^. If the results of Experiment 2 were indeed due to observers making comparisons between stimuli of the same category, contextual stimuli belonging to different categories should prevent such comparisons and preserve redundancy masking. This was indeed the case: in Experiment 3, in which the line triplet was presented with *dissimilar* contextual stimuli, observers reported perceiving a line pair in the majority of trials (68%). Since the stimulus range was the same (1–5) in both experiments, we can rule out the stimulus range itself as an explanation. Similarly, we did not detect any encoding of the ensemble statistics of stimulus range (e.g., regression towards the mean^21^). Rather, the same stimulus range elicited different numerosity perceptions depending on the spatial features of the stimulus set.

Other perceptual effects that could have affected our results are adaptation^59,60^ and priming^61–64^. While jittering of stimulus positions and random presentation to the left or right of fixation should have prevented major influences of adaptation on the perceived number of items, differences of adaptation in the *similar* and *dissimilar* condition cannot be completely excluded. However, any effects of adaptation on the perceived number of lines in the line triplet would have been stronger in the *similar* than in the *dissimilar* condition, with a corresponding expected reduction of the number of perceived items in the *similar* condition. Instead, the number of perceived lines was clearly larger in the *similar* than in the *dissimilar* condition. Similarly, the improvement of target perception through priming by a preceding stimulus^61^ could have had different effects in the *similar* and *dissimilar* conditions. Specifically, as the two conditions did not differ in regard to the number of presented items, not priming by the *number* of items per se, but stronger priming by similar than dissimilar items would be required (e.g.^65^). The relatively small number of trials in which primes could have actually improved performance, and the overestimation of the number of lines in the triplet in Experiment 2 and underestimation in Experiment 3, however, suggest a rather small effect of priming on the results. However, there was an effect of the previous trial on estimates in the subsequent trial: the larger the number of items in the preceding trial (i.e., one trial back) the larger was the enumeration response to the line triplet. Similar dependence on previous trials were shown for larger numbers of items (in the numerosity estimation range), but not in the subitizing range^26^. However, as this effect occurred in both the *similar* and *dissimilar* condition, modulating only the extent but not the direction of deviation scores of the line triplet (positive in the similar, negative in the dissimilar condition), it falls short to explain the difference between the two conditions. Overall, to measure minimally biased appearance and diminish the potential influence of confounding factors, we suggest that *neutral* or *dissimilar* contextual stimuli have advantages compared to *similar* contextual stimuli, and may shed light on aspects of peripheral stimulus appearance that otherwise go unnoticed.

We investigated the influence of a standard set of contextual stimuli presented in temporal proximity with the target and compared it with minimally biasing contexts. Revealing a substantial difference of overestimation versus underestimation, our results showed that redundancy masking was concealed in a typical set of contextual stimuli. Because experiments on visual perception usually use stimulus sets containing highly similar items, strong perceptual effects such as redundancy masking could remain overlooked. Our results highlight the potentially profound impact of contextual stimuli on empirical results and urge caution in designing experiments to be aware of biases that might conceal key aspects of target perception.

## Materials and methods

### Experiment 1: neutral context

#### Participants

55 voluntary participants (age range: 19–56, 22 male) who were naïve to the aim of the study completed a single trial. All observers reported normal or corrected-to-normal visual acuity. The experiment was performed in public premises at the University of Bern. All the experimental protocols were approved by the local ethics committee at the University of Bern. All procedures were in accordance with the Declaration of Helsinki.

#### Stimuli and procedure

Stimuli were generated with Psychopy v2.7.11^66^ and displayed on a 12.5″ laptop with a resolution of 1366 × 768 and a refresh rate of 60 Hz. Observers binocularly viewed the monitor from a distance of approximately 35 cm. A black disc (diameter = 0.2°; 2 cd/m^2^) at the center of the screen served as the fixation point. Stimuli consisted of three black (2 cd/m^2^) lines that were 1.1° in length and 0.04° in width, presented on a uniform grey background (80 cd/m^2^). The lines were arranged radially with respect to fovea (Fig. 1a). The center-to-center spacing between adjacent lines was 0.78° (shown previously to be well above the resolution limit of typical observers^30^). The line triplet was centered at 10° eccentricity in the right visual field (observers were told that the stimulus could appear either to the right or left of the fixation disc).

Observers performed only a single trial. They had no prior knowledge about any characteristics of the stimulus. Before stimulus presentation, participants were asked to fixate the fixation disc. Next, the experimenter initiated the trial with a key press. The stimulus was presented for 150 ms. After the stimulus presentation, the experimenter asked observers to freely report what they perceived. If their response did not contain any details regarding the number of items (e.g., ‘I saw black lines’), participants were asked how many items they perceived. Responses were recorded by the experimenter.

#### Analysis

In all experiments, performance was defined as the number of items presented subtracted from the number of items reported (“deviation”). Hence, if the number of items reported was the same as the number of items presented, the deviation was zero; reporting more items than presented yielded deviation scores above zero, and reporting fewer items than presented yielded deviation scores below zero. Due to a violation of the normality assumption, a one-sample sign test was used to assess if the number of lines reported differed from the number of lines presented.

### Experiment 2: similar context

#### Participants

Five undergraduate students (age range: 19–27, one male) from the University of Bern participated in the experiment in exchange for course credit. All observers reported normal or corrected-to-normal visual acuity. Observers were naïve regarding the aim of the study. All study participants signed a consent form and were informed about the general procedure. The number of participants recruited in Experiment 2 and 3 was determined based on our earlier study^30^.

#### Stimuli and procedure

Stimuli were generated with Psychopy v2.7.11^66^ and displayed on a 21″ CRT monitor with a resolution of 1024 × 768 and a refresh rate of 75 Hz. The experiment was conducted in a dimly illuminated room. Observers binocularly viewed the monitor from a distance of 57 cm, and were supported by a chin and head rest. A black disc (diameter = 0.2°; 2 cd/m^2^) at the center of the screen served as a fixation point throughout the experiment. Stimuli consisted of black (2 cd/m^2^) lines that were 1.1° in length and 0.04° in width, presented on a uniform grey background (80 cd/m^2^). As in Experiment 1, the lines were arranged radially. The number of presented lines ranged from 1 to 5. The center-to-center spacing between adjacent lines within a line array was identical, but varied randomly across trials to preclude the use of spacing and overall extent as cues (see example stimuli in Fig. 2a). We used spacings of 0.39°, 0.58°, and 0.78°, yielding maximum extents of the line arrays of 0.39°, 0.58°, and 0.78° when 2 lines are presented; 0.78°, 1.16°, and 1.55° when 3 lines are presented; 1.16°, 1.74°, and 2.33° when 4 lines are presented; 1.55°, 2.33°, and 3.1° when 5 lines were presented, respectively. The line array was centered at 10° eccentricity to either the right or the left of the fixation disc. The position of the line array was slightly varied at random across trials (centered at 10° or jittered 0.08° either up, down, left or right).

At the beginning of the experiment, the fixation disc was presented for 1 s. Next, a stimulus was presented for 150 ms to the left or the right of fixation. Observers were required to indicate the number of lines they perceived with a key press on the number pad (0–9). Response time was unconstrained. The next stimulus was presented 440 ms after each response. The stimulus location (left or right of fixation), the number of lines (1 to 5), and the spacings (0.39°, 0.58°, and 0.78°) were randomized within each block. Observers completed 2 blocks with 180 trials (72 for each number of lines). Figure 2 illustrates a trial sequence (2a) and a schematic depiction of the procedure (2b).

Before the experiment, for each participant we verified that the spacing between adjacent lines was above their resolution limit. A two-line discrimination task was performed at the farthest eccentricities of lines in the main experiment: two lines with varying spacings were presented at 10.8°, 11.2°, and 11.6° degrees (corresponding to the farthest eccentricities for spacings of 0.39°, 0.58°, and 0.78°, respectively). At each eccentricity, five different spacings were presented (3 eccentricities × 5 spacings × 10 trials; 150 trials in total). Participants were asked whether they perceived one or two lines. All observers reported two lines in 100% of the trials with the spacings (0.39°, 0.58°, and 0.78°) presented in the main experiment.

#### Analysis

As evidenced by our previous investigations of redundancy masking^30,31^, we did not expect a monotonic relationship between deviation scores and the number of items presented in all conditions. Thus, we analyzed the deviation scores with linear mixed-effects models specifying subjects as a random factor, and the number of lines presented as the fixed effect. For model selection, null models (without the fixed effect) and full models (with the fixed effect) were fitted and hierarchically compared. Similar incremental model building was used to select the minimum degree polynomial that fitted the data. Likelihood-ratio tests with Satterthwaite's approximation for the degrees of freedom were performed for model comparisons, and the Akaike information criterion was used to select the best fitting model^67^. Confidence intervals were calculated with the ggpredict function of the ggeffects package^68^. The model *R* squared statistic (*R*^2^) was computed to quantify goodness-of-fit with the r2glmm package using the Nakagawa and Schielzeth approach^69^. Assumptions underlying the models were checked with diagnostic plots of residuals^70^. Shapiro–Wilk tests were used to test for deviations from normality in the residuals. A third-degree polynomial regression was used to fit the deviation scores on the number of lines presented (*R*^2^ = 0.28). The random effect structure included random slopes and random intercepts for each subject. As our main stimulus of interest is the line triplet (Experiment 1) for which redundancy masking was expected to be most pronounced, the analyses in Experiment 2 and 3 focus on the line triplet as well, allowing direct comparisons between the similar and dissimilar contextual conditions. Thus, a one-sample t-test was used to assess if the number of lines reported differed from the number of lines in the line triplet. Cohen’s *d* is reported for the measure of effect size (the same test and measure of effect size were applied for the line triplet in Experiment 3).

### Experiment 3: dissimilar context

#### Participants

Six students (age range 20–23, one male) participated in the experiment in exchange for course credit. None of the observers had participated in Experiment 2.

#### Stimuli and procedure

The apparatus was the same as in Experiment 2. The line triplet (using only the 0.39° spacing) was also the same as in Experiment 2. The contextual stimuli consisted of various items: the line triplet, arrays of symbols and letters (“&”, “ < ”, “ > ”, “A”, “B”, “G”), geometrical shapes (circles, hexagons, triangles), and stacked horizontal lines. The letters and symbols were from Arial Narrow font, and 0.85° in height (varying slightly depending on the item). The shapes were 0.78° in height and width. The size of the horizontal lines was the same as of the line triplet (1.1° in length and 0.04° in width). All items were black (2 cd/m^2^). The stimuli other than the line triplet were arranged either tangentially (with respect to fovea) or in different canonical configurations (triangle, square, and quincunx). The center-to-center spacing between adjacent items arranged tangentially was 1.55° (except for the horizontal lines: 0.78°, to prevent visual comparisons with the line triplet). The center-to-center spacings between adjacent items varied depending on the arrangement (from 1.1° to 1.55° in triangular arrangements, 1.6° to 3.3° in square and quincunx arrangements). All trials with two items were presented in tangential arrangements. Half of the trials with three items consisted of the (radially arranged) line triplet, and the other half consisted of random shapes in either tangential or triangular arrangements. The trials with four items were randomly presented in either tangential, square, or rotated square (‘diamond’) arrangements. Half of the trials with five items was presented in tangential and half in quincunx arrangements. Example stimuli featuring different items are illustrated in Fig. 3a.

The task and procedure were the same as in Experiment 2 with a few differences. The stimulus location (left or right of fixation), the number of items (1 to 5), and item type were randomized within each block. Observers completed 2 blocks consisting of 100 trials. The line triplet was presented in 10 trials per block (20 trials in total; previously, redundancy masking was shown to be robust with a single trial of unlimited presentation time^29^, and with 24 trials/condition in a paradigm similar to the one used here^30^). The other items were presented in the remaining 90 trials (20 trials for 1, 2, 4, 5 items; and 10 trials for the other triplets). Hence, each number of items was presented the same number of times (in total 40 times each). Figure 3 illustrates a trial sequence (3a) and a schematic depiction of the procedure (3b).

As in Experiment 2, a two-line discrimination task (with 100 trials) was performed before the main experiment: two lines with varying spacings were presented at the farthest eccentricity of the main experiment (10.8°). All observers reported two lines in 100% of the trials with the spacing (0.39°) presented in the main experiment.

#### Analysis

The analyses were the same as in Experiment 2 except for the following differences. The deviation scores for the 10 types of items (excluding the line triplet) were averaged for each number of items presented. The deviation scores for these items were analyzed by linear mixed-effects models specifying subject as a random factor, and the number of items presented as the fixed effect. A linear regression was used to fit the deviation scores on the number of items presented (*R*^2^ = 0.25). The random effect structure contained random slopes for each subject.

## Supplementary Information

Supplementary Information.

## Data Availability

The datasets generated during the current study are available in the BORIS (Bern Open Repository and Information System) repository: https://boris.unibe.ch/id/eprint/145214.
