# Supplementary material for: Hidden by bias: how standard psychophysical procedures conceal crucial aspects of peripheral visual appearance
Source: Sci Rep. 2021 Feb 18;11:4095. doi: 10.1038/s41598-021-83325-7 (PMC7892995; doi:10.1038/s41598-021-83325-7)
Supplement: Supplementary file 1 — Supplementary Information. [file 41598_2021_83325_MOESM1_ESM.pdf]

Supplementary material for

Hidden by bias: how standard psychophysical procedures conceal crucial aspects of peripheral visual appearance

Fazilet Zeynep Yildirim[1]*, Daniel R. Coates[1, 2], Bilge Sayim[1, 3]

[1]Institute of Psychology, University of Bern, Fabrikstrasse 8, 3012 Bern, Switzerland.

[2]College of Optometry, University of Houston, Houston, TX 77204, USA.

[3]SCALab - Sciences Cognitives et Sciences Affectives, CNRS, UMR 9193, University of Lille, 59000 Lille, France.

*Corresponding author: Fazilet Zeynep Yildirim

Email: fazilet.yildirim@psy.unibe.ch

Address: Fabrikstrasse 8, 3012, Bern, Switzerland

Telephone: +41 31 631 38 05

ORCIDs

Fazilet Zeynep Yildirim: https://orcid.org/0000-0002-8754-8137

Daniel R. Coates: https://orcid.org/0000-0001-5682-2554

Bilge Sayim: https://orcid.org/0000-0002-7589-5385

Keywords

Redundancy masking, stimulus set, contextual effects, peripheral vision, appearance

This PDF file includes:

Supplementary figure 1 and 2

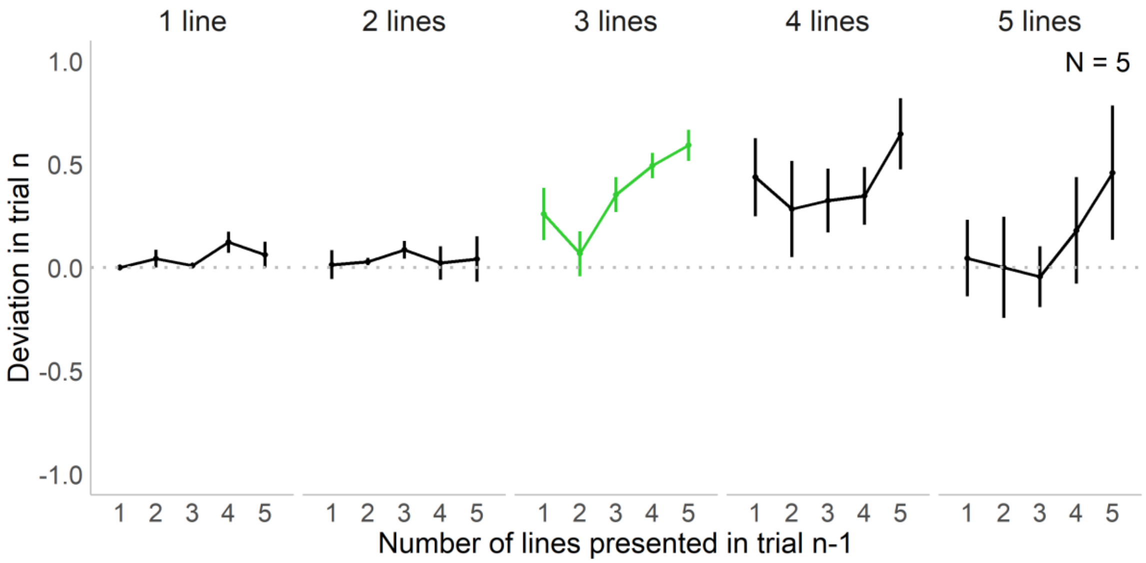

Supplementary Figure S1. Average deviation scores (in trial n) as a function of the number of lines presented in the previous trial (trial n-1) for Experiment 2. Deviation scores were calculated by subtraction of the number of lines presented from the number of lines reported. Black and green data points show mean deviation scores ($\pm SEM$) for 1-5 lines, and 3 lines (the line triplet), respectively. Generalized linear mixed-effects models were used to analyze the data, specifying subjects as the random factor, and the number of lines presented in trial n-1, and the number of lines presented in trial n as the fixed effects. A third-degree polynomial regression with an interaction term showed that the main effects of the number of lines presented in trial n-1 (X^2 (2) = 30.51, p = 2.376e-07), the number of lines presented in trial n (X^2 (3) = 62.10, p = 2.092e-13), and the interaction effect between the two were significant (X^2 (6) = 15.48, p = .017). Response latencies across observers were on average 0.98 s ± SD 0.37. Hence, the perceived number of lines was attracted toward the stimulus seen about 1.42 s (mean response time 0.98 s + ISI 0.44 s) ago.

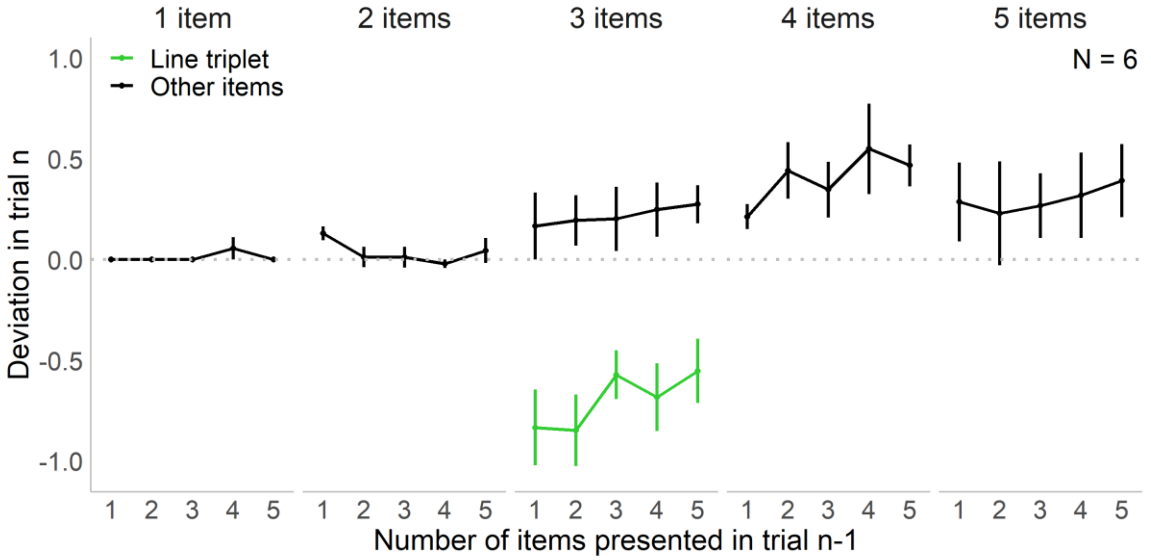

Supplementary Figure S2. Average deviation scores (in trial n) as a function of the number of items presented in the previous trial (trial n-1) for Experiment 3. Deviation scores were calculated by subtraction of the number of items presented from the number of items reported. Black and green data points show mean deviation scores (±*SEM*) for 1-5 items, and 3 lines (the line triplet), respectively. For the line triplet, generalized linear mixed-effects models were used to analyze the data, specifying subjects as the random factor, and the number of lines presented in trial n-1 as the fixed effect. A linear regression showed a significant main effect of the number of items presented in trial n-1 (X^2 (1) $= 7.00$, $p = .0081$). For the other items, again, generalized linear mixed-effects models were used, specifying subjects as the random factor, and the number of items presented in trial n-1, and the number of items presented in trial n as the fixed effects. A linear regression without an interaction term showed no significant main effect of the number of items presented in trial n-1 (X^2 (1) $= 1.63$, $p = .20$), but a significant main effect of the number of items presented in trial n (X^2 (1) $= 5.59$, $p = .018$). Response latencies across observers were in average 1.17 s ± *SD* 0.28. Hence, the perceived number of lines was attracted toward the stimulus seen about 1.61 s (mean 1.17 s+ ISI 0.44 s) ago.
